# Supplementary material for: Identification of photoperiod-induced specific miRNAs in the adrenal glands of Sunite sheep (Ovis aries)
Source: Front Vet Sci. 2022 Jul 22;9:888207. doi: 10.3389/fvets.2022.888207 (PMC9354845; doi:10.3389/fvets.2022.888207)
Supplement: Supplementary file 1 [file Data_Sheet_1.ZIP › Supplementary file/miRDeep2 file.zip!/miRDeep2 file/Know_Structure/LP42A2/oar-mir-544.pdf]

miRBase precursor : oar-mir-544
Total read count : 469
oar-miR-544-5p read count : 433
oar-miR-544-3p read count : 29
remaining reads : 7

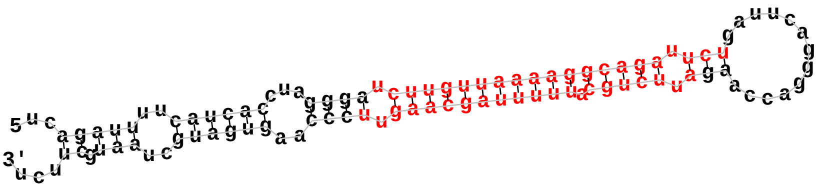

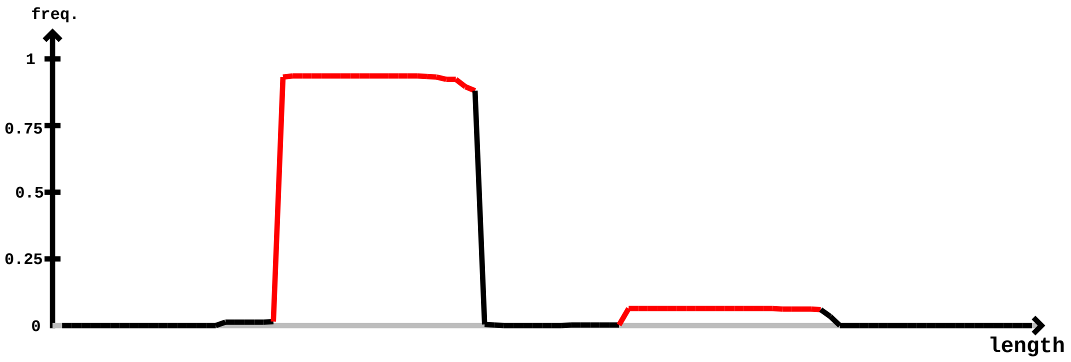


```
                                 oar-miR-544-5p
                                                                             oar-miR-544-3p
5'- ucagauuuucaucaccuagggaucuuguuaaaaggcagauucugauucagggaccaagauucugcauuuuuagcaaguucccaagugaugcuaaugcuucu  -3'   exp
    ..((((..((((((...((((.(((((((((((((((((.(((.............))).))))).))))))))))).))))..))))))..))).))...  reads   mm   sample
    ................uagggaucuuguuaaaaggca................................................................  1       0    seq
    ................uagggaucuuguuaaaaggcag...............................................................  1       0    seq
    ................uagggaucuuguuaaaaggcaga..............................................................  4       0    seq
    .....................aucuuguuaaaaggcagauucu..........................................................  1       0    seq
    ......................uAuuguuaaaaggcagauu............................................................  1       1    seq
    ......................ucuuguuaaaaggcagauu............................................................  12      0    seq
    ......................ucuuguuaaaaggcagauuc...........................................................  4       0    seq
    ......................ucuuguuaaaaggcagauuU...........................................................  3       1    seq
    ......................ucuuguuaaaaggcagGuucu..........................................................  1       1    seq
    ......................ucuuguuaaaaggcagauucu..........................................................  366     0    seq
    ......................ucuuAuuaaaaggcagauucu..........................................................  2       1    seq
    ......................ucuuguuaaaaggcagauuUu..........................................................  1       1    seq
    ......................ucuuguuaaaaggcagauucA..........................................................  21      1    seq
    ......................ucuuguuaaaaggcagaCucu..........................................................  2       1    seq
    ......................ucuuguuUaaaggcagauucu..........................................................  1       1    seq
    ......................ucuuguuaCaaggcagauucu..........................................................  1       1    seq
    ......................ucuuguuaGaaggcagauucu..........................................................  4       1    seq
    ......................ucuuCuuaaaaggcagauucu..........................................................  1       1    seq
    ......................uUuuguuaaaaggcagauucu..........................................................  2       1    seq
    ......................Ccuuguuaaaaggcagauucu..........................................................  5       1    seq
    ......................ucuuguCaaaaggcagauucu..........................................................  1       1    seq
    ......................ucuuguuaaaaggcagauucug.........................................................  1       0    seq
    ......................ucuuguuaaaaggcagauucuAa........................................................  1       1    seq
    .......................cuuguuaaaaggcagauucu..........................................................  1       0    seq
    .......................cuuguuaaaaggcagauucA..........................................................  1       1    seq
    ....................................................accaagauucugcauuuuuagc...........................  1       0    seq
    ..........................................................auucugcauuuuuagcaagC.......................  1       1    seq
    ..........................................................auucugcauuuuuagcaaguu......................  11      0    seq
    ..........................................................auucugcauuuuuagcaagCu......................  1       1    seq
    ..........................................................auucugcauuuuuagcaaguuU.....................  7       1    seq
    ..........................................................auucugcauuuuuagcaaguuc.....................  9       0    seq
```
